# Declining recurrence among ductal carcinoma in situ patients treated with breast-conserving surgery in the community setting

**DOI:** 10.1186/bcr2453

**Published:** 2009-11-18

**Authors:** Laurel A Habel, Ninah S Achacoso, Reina Haque, Larissa Nekhlyudov, Suzanne W Fletcher, Stuart J Schnitt, Laura C Collins, Ann M Geiger, Balaram Puligandla, Luana Acton, Charles P Quesenberry

**Affiliations:** 1 Division of Research, Kaiser Permanente, Northern California, 2000 Broadway, Oakland, CA 94612, USA; 2 Research and Evaluation, Kaiser Permanente, Southern California, 100 South Los Robles, Pasadena, CA 91101, USA; 3 Department of Population Medicine, Harvard Medical School/Harvard Pilgrim Health Care Institute, 133 Brookline Avenue, Boston, MA 02215, USA; 4 Division of Medicine, Harvard Vanguard Medical Associates, 133 Brookline Avenue, Boston, MA 02215, USA; 5 Department of Pathology, Beth Israel Deaconess Medical Center and Harvard Medical School, 330 Brookline Avenue, Boston, MA 02215, USA; 6 Division of Public Health Sciences, Wake Forest University School of Medicine, Medical Center Boulevard, Winston-Salem, NC 27157, USA; 7 Department of Pathology, Kaiser Permanente Medical Center, 280 West MacArthur Boulevard, Oakland, CA 94611, USA

## Abstract

**Introduction:**

Randomized trials indicate that adjuvant radiotherapy plus tamoxifen decrease the five-year risk of recurrence among ductal carcinoma in situ patients treated with breast-conserving surgery from about 20% to 8%. The aims of this study were to examine the use and impact of these therapies on risk of recurrence among ductal carcinoma in situ patients diagnosed and treated in the community setting.

**Methods:**

We identified 2,995 patients diagnosed with ductal carcinoma in situ between 1990 and 2001 and treated with breast-conserving surgery at three large health plans. Medical charts were reviewed to confirm diagnosis and treatment and to obtain information on subsequent breast cancers. On a subset of patients, slides from the index ductal carcinoma in situ were reviewed for histopathologic features. Cumulative incidence curves were generated and Cox regression was used to examine changes in five-year risk of recurrence across diagnosis years, with and without adjusting for trends in use of adjuvant therapies.

**Results:**

Use of radiotherapy increased from 25.8% in 1990-1991 to 61.3% in 2000-2001; tamoxifen increased from 2.3% to 34.4%. A total of 245 patients had a local recurrence within five years of their index ductal carcinoma in situ. The five-year risk of any local recurrence decreased from 14.3% (95% confidence interval 9.8 to 18.7) for patients diagnosed in 1990-1991 to 7.7% (95% confidence interval 5.5 to 9.9) for patients diagnosed in 1998-1999; invasive recurrence decreased from 7.0% (95% confidence interval 3.8 to 10.3) to 3.1% (95% confidence interval 1.7 to 4.6). In Cox models, the association between diagnosis year and risk of recurrence was modestly attenuated after accounting for use of adjuvant therapy. Between 1990-1991 and 2000-2001, the proportion of patients with tumors with high nuclear grade decreased from 46% to 32% (*P *= 0.03) and those with involved surgical margins dropped from 15% to 0% (*P *= 0.03).

**Conclusions:**

The marked increase in the 1990s in the use of adjuvant therapy for ductal carcinoma in situ patients treated with breast-conserving surgery in the community setting only partially explains the 50% decline in risk of recurrence. Changes in pathology factors have likely also contributed to this decline.

## Introduction

The diagnosis of ductal carcinoma in situ (DCIS) has increased several-fold since the early 1980s [1,2], due mainly to the increase in mammographic screening and the greater tendency to biopsy radiographically suspicious lesions. Previously, DCIS constituted 1 to 5% of breast cancer diagnoses and was usually detected as a palpable mass. DCIS currently accounts for up to 20% of the breast cancers diagnosed in screened populations [3] and is frequently microscopic and asymptomatic [4]. It is estimated that approximately 58,000 new cases of DCIS were diagnosed in the U.S. in 2008 [5].

Before the 1980s, virtually all breast cancer, in situ as well as invasive, was treated with mastectomy. In the mid-1980s, clinical trials demonstrated that breast-conserving surgery (BCS) is an appropriate treatment strategy for most early invasive breast cancer [6,7]. Consequently, use of BCS was also considered appropriate for most patients with DCIS. Up to 70% of DCIS patients in the U.S. are currently being treated with BCS, although use of BCS varies by geographic region and is highest in the northeast and west [8,9].

Currently recommended breast-conserving regimens for DCIS include breast-conserving surgery (BCS) alone, BCS plus radiotherapy, and BCS plus radiotherapy and tamoxifen [10,11]; however, there is no consensus on which women should be treated with the different regimens. No other adjuvant therapies are approved for DCIS, although some, such as aromatase inhibitors, are being evaluated in clinical trials [12].

Among DCIS patients treated with BCS, use of adjuvant radiotherapy has increased substantially over the last two decades [2,8,9]. More recently, the use of tamoxifen, which was approved by the U.S. Federal Drug Administration (FDA) as adjuvant therapy for DCIS in 2000 [13], has also increased in this population [14]. The extent to which other therapies, such as aromatase inhibitors, are being used off-label is unclear.

Cancers considered to be recurrent disease by a clinician are not captured well by most cancer registries, such as the National Cancer Institutes' Surveillance, Epidemiology, and End Results (SEER) program of registries, which only captures new primary cancers. Consequently, there are few population-based data available on risk of local recurrence following BCS for DCIS. Data from randomized trials indicate that treatment with adjuvant radiotherapy decreases local recurrences by about 50% [15-17]. Although clinical trial data have been somewhat conflicting [18,19], it appears that adding tamoxifen to BCS plus radiotherapy may decrease local recurrences by another 50% [18]. Overall, the addition of these two adjuvant therapies to BCS alone appears to reduce the five-year local recurrence rate (combination of DCIS and invasive disease) from 20% to approximately 8% [15,19]. However, we do not know if results of randomized clinical trials of treatment effects are generalizable to the community setting. To our knowledge, data are currently unavailable on whether rates of local recurrence in the community setting have changed as the use of these adjuvant therapies for DCIS has increased.

The aims of this study were to determine: 1) trends in adjuvant treatment among DCIS patients diagnosed between 1990 and 2001 and treated with BCS in three community-based health care plans, and 2) five-year risk of local recurrence or any second breast cancer event among those diagnosed between 1990 and 1999. We also examined the extent to which treatment with radiotherapy and tamoxifen could explain observed time trends in risk of recurrence. Finally, we determined whether there have been changes in histopathologic factors that could have contributed to observed trends.

## Materials and methods

The study was conducted under the auspices of the National Cancer Institute-funded Cancer Research Network (CRN), a consortium of 14 health maintenance organizations (HMOs) with more than 12 million enrollees. The overall goal of the CRN is to increase the effectiveness of preventive, curative, and supportive interventions for major cancers through a program of collaborative research, and to determine the effectiveness of cancer control interventions that span the natural history of major cancers among diverse populations and health systems.

### Study population

We identified all patients diagnosed with a first primary unilateral DCIS between 1990 and 2001 and treated with BCS at three HMOs participating in the CRN: Kaiser Permanente of Northern California (KPNC), Kaiser Permanente of Southern California (KPSC), and Harvard Pilgrim Health Care (HPHC).

Patients were eligible if they were less than age 85 years at diagnosis and had no prior invasive cancer (breast or other site). Patients were excluded if breast cancer (DCIS or invasive disease) was diagnosed in the contralateral breast at the time of the index DCIS diagnosis or if they had a mastectomy within six months of their index DCIS. Patients were also excluded if medical care (treatment or follow-up) for their DCIS was obtained from providers outside of the three health plans.

### Data sources

Cancer registries were used to identify patients with an initial diagnosis of DCIS at KPNC and KPSC. These registries provide data on new primary cancers to the SEER program and include information on birth date, race/ethnicity, prior cancer diagnoses, laterality of the index DCIS, type of surgical treatment, treatment with radiotherapy, and treatment with hormonal therapy. At HPHC, electronic medical records and claims codes were used to identify DCIS diagnoses and surgical treatment.

At each of the three health plans, medical records of potentially eligible patients were reviewed to confirm the initial diagnosis, treatment, and laterality of the index DCIS and to obtain information on subsequent breast cancer events. Information was also collected on history of breast cancer, surveillance mammography, and on all subsequent breast biopsies. In addition, data were abstracted on several patient and clinical factors at the time of their index DCIS (for example, method of detection, family history of breast cancer in first degree relative, height, weight).

To explore whether several pathologic features considered to be risk factors for recurrence after DCIS [20-26] may have changed over time and contributed to changes in risk of recurrence, we examined their distributions by diagnosis year among patients included in a case-control study nested within this same DCIS cohort. This case-control study included all recurrences (cases). At each case's recurrence, up to two patients (controls) were randomly selected from cohort members still under follow-up and without a recurrence (that is, incidence density sampling [27]). Controls were individually matched to their case on health plan, age at diagnosis (<45, 45 to 54, 55 to 64, 65 to 84 years), and calendar year of diagnosis (1990-1991, 1992-1993, 1994-1995, 1996-1997, 1998-1999, and 2000-2001).

Diagnostic slides were available on 297 cases and 496 controls, and a standardized central histopathology review was conducted by expert DCIS pathologists (authors SJS, LCC) that included confirmation of the initial DCIS diagnosis (cases = 245, controls = 416) and the assessment of multiple features including evaluation of surgical margins, and among those with uninvolved margins, width of margin. A margin was scored as involved if there was DCIS at the inked tissue edge and as *close *if there was DCIS within 1 mm of the inked margin. The extent of margin involvement was recorded as a linear value, but also as the number of low power microscopic fields of DCIS at or close to the inked margin. Other features included tumor size, predominant nuclear grade, necrosis (comedo or punctate) and primary architectural pattern (comedo, solid, papillary, micropapillary, cribriform or clinging), among others.

Tumor blocks were retrieved on 210 cases (89%) and 364 controls (88%) and sent to PhenoPath Laboratories (Seattle, WA) for estrogen receptor (ER) immunostaining (rabbit monoclonal (clone SP1), Lab Vision) and scoring. Immunostains were performed on four to five-micron sections cut from a single paraffin block and appropriate positive and negative controls were included in each staining run. The scoring was conducted by visually estimating the percentage of ER-positive nuclei in the tumor population. Those with <1% staining were scored as 0 (negative); the positives were scored as 1 to 25% = 1+, 25 to 75% = 2+, >75% = 3+.

### Endpoints

Local recurrence was our primary endpoint of interest and was defined as DCIS or invasive breast cancer in the involved or ipsilateral breast at least six months after the index diagnosis. Women with a local recurrence included those with an ipsilateral breast tumor only as well as those who had ipsilateral disease with regional or distant involvement. Secondary analyses were conducted for: 1) any second breast cancer event, which included local recurrences, contralateral breast cancers and regional or distant breast metastases (with or without local involvement) and 2) contralateral disease only, since tamoxifen also has been shown to reduce the risk of these endpoints [18].

DCIS or invasive disease identified during the first six months after the index DCIS was considered part of the initial disease. Therefore, women with invasive breast cancers identified during this period were excluded from the cohort as they were considered to have invasive disease, and not pure DCIS, at presentation.

### Statistical analyses

Follow-up began at six months after diagnosis of DCIS and ended at date of recurrence, prophylactic mastectomy of the ipsilateral breast, contralateral breast cancer, non-breast invasive cancer, death, or last chart note, whichever came first. All endpoints were defined with respect to the first event following DCIS diagnosis and did not include breast cancer events that occurred after the first subsequent cancer. Five-year risk estimates (any local recurrence, local invasive recurrence, any second breast cancer, any second invasive breast cancer, contralateral breast cancer) were generated [28,29], stratified by diagnosis year and, for some analyses, by treatment. These analyses were restricted to diagnosis years 1990 to 1999 so that patients would have the opportunity for five years of follow-up (most follow-up ended in 2004). In addition, Cox regression modeling was used to estimate relative risks for recurrence associated with diagnosis year (1990 to 1999), both with and without adjustment for treatment with radiotherapy and tamoxifen [30]. We also examined potential confounding by age, race, family history of breast cancer, body mass index (BMI), method of detection of the initial DCIS, and re-excision or post-surgical mammogram in the three months after initial biopsy with DCIS. Time since diagnosis of the index DCIS was the time scale in these Cox models. These Cox model analyses censored follow-up time at five years to be consistent with the risk estimates. Results for analyses with and without censoring at five years were similar, and so we have only presented the censored results. We tested the validity of the proportional hazards assumption by adding an interaction term between diagnosis year and time, and there was no evidence of non-proportionality.

It is important to note that because the standardized pathology review was conducted only on cases and controls and because cases and controls were matched on diagnosis year, it was not possible to adjust diagnosis year estimates for pathologic variables. F-tests were performed to examine changes over time.

### Institutional Review Board approval

The study was approved by the Kaiser Permanente Inter-regional Institutional Review Board (for KPNC and KPSC) and by the Institutional Review Boards at Harvard Pilgrim Health Care and Beth Israel Deaconess Medical Center.

## Results

Of the 3,668 patients identified as potentially eligible by our cancer registries or electronic medical records, there were 520 patients who were determined by chart review to be ineligible for one or more of the following reasons: miscoded as having DCIS in the tumor registry or diagnosed with invasive breast cancer within six months of index DCIS (n = 97), synchronous cancer in the uninvolved or contralateral breast (n = 29), prior breast cancer (n = 91), prior invasive cancer at another site (n = 125), 85 years of age or older at diagnosis (n = 15) or had less than six months of follow-up (mastectomy within six months (n = 96), death within six months (n = 6), or not a member at diagnosis or left the health plan within six months (n = 92)). The eligibility of 82 patients could not be determined because of incomplete or unavailable medical records and they were considered lost to the study. Also, 29 did not have complete information on adjuvant therapy. Of the 3,037 women determined to be eligible by chart review, 42 had no pathology report confirming breast-conserving surgery, leaving 2,995 patients available for this study.

### Characteristics of the final cohort

Of the 2,995 eligible DCIS patients, 325 (10.9%) had a recurrence as a first cancer event during a median follow-up of 4.8 years (range 0.5 to 15.7 years). Of these recurrences, 294 were confined to the ipsilateral breast, and 31 had an ipsilateral breast tumor plus regional or distant involvement. An additional nine patients had regional/distant disease without evidence of an ipsilateral tumor. Another 129 DCIS patients had a subsequent cancer in the contralateral breast as a first event during follow-up. There were 18 women whose follow-up time was censored at the time of prophylactic mastectomy of the ipsilateral breast, 133 at diagnosis of non-breast invasive cancer, 69 at death, and 2,312 at last chart note.

The number of patients with an index DCIS diagnosis in 2000-2001 was almost triple that in 1990-1991 (Table 1), reflecting the increasing diagnosis rates as well as the increasing use of BCS over time. The majority of DCIS patients were white, but approximately a third were minorities; 25% were younger than age 50 years, and 19% were age 70 years or older at the index DCIS diagnosis. Approximately 43% of patients were treated with BCS alone, 42% with BCS plus radiotherapy, 11% with BCS plus radiotherapy and tamoxifen, and less than 5% with BCS plus tamoxifen.

**Table 1:** Selected characteristics of DCIS^a ^study cohort

Characteristics	Full cohort (Number)	%	Recurrences (Number)	%
**Total**	2995	100.0	325	100.0

**Diagnosis year**				

1990-1991	256	8.6	54	16.6

1992-1993	354	11.8	50	15.4

1994-1995	444	14.8	76	23.4

1996-1997	558	18.6	81	24.9

1998-1999	663	22.1	47	14.5

2000-2001	720	24.0	17	5.2

**Age at diagnosis (years)**				

<50	767	25.6	103	31.7

50-59	853	28.5	94	28.9

60-69	800	26.7	86	26.5

70+	575	19.2	42	12.9

**Race**				

Asian	360	12.0	29	8.9

Black	289	9.6	47	14.5

Hispanic	257	8.6	28	8.6

Other	8	0.3	0	0.0

White	2045	68.3	220	67.7

Unknown	36	1.2	1	0.3

**Adjuvant therapy**				

None (BCS^b ^only)	1275	42.6	223	68.6

Radiotherapy (no Tamoxifen)	1257	42.0	95	29.2

Tamoxifen (no Radiotherapy)	132	4.4	2	0.6

Radiotherapy+Tamoxifen	331	11.0	5	1.5

^a ^Ductal carcinoma in situ

^b ^Breast-conserving surgery

### Treatment trends

**Figure 1:**
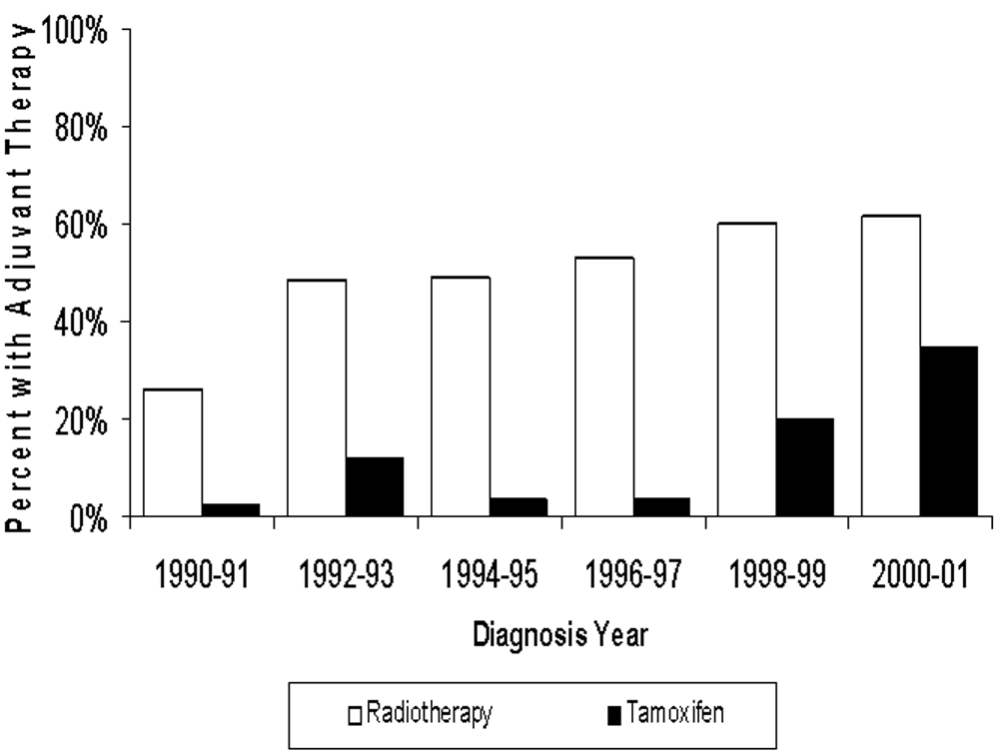
Adjuvant treatment by diagnosis year. The white bars indicate the percent of women treated with radiotherapy (with or without tamoxifen); the black bars indicate the percent of women treated with tamoxifen (with or without radiotherapy).

Among DCIS patients treated with BCS, the use of adjuvant radiotherapy increased from 25.8% for patients diagnosed in 1990-1991 to 61.3% for those diagnosed in 2000-2001 (Figure 1). Treatment with adjuvant tamoxifen increased from 2.3% to 34.4% during this same period.

### Risk of local recurrence and other breast cancer events at five years

Of the 325 local recurrences, 245 occurred within the first five years. Within five years, there were a total of 358 patients with any second breast cancer (245 with local recurrence, 28 with regional or distant recurrences with or without local involvement and 85 with contralateral cancer).

**Figure 2:**
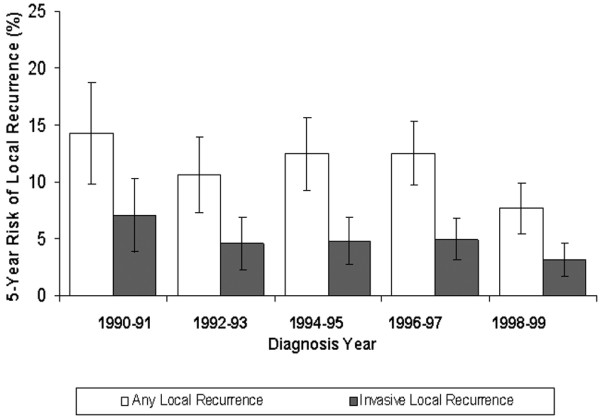
Five-year risk of local recurrence by diagnosis year. The white bars indicate the risk of any local recurrence; the grey bars indicate the risk of an invasive local recurrence. The length of the vertical line through the bar indicates the 95% confidence interval (CI).

The five-year risk of a local recurrence (DCIS or invasive) decreased from 14.3% (95% CI 9.8 to 18.7) for patients diagnosed in 1990-1991 to 7.7% (95% CI 5.5 to 9.9) for patients diagnosed in 1998-1999 (Figure 2). The risk of an invasive local recurrence decreased from 7.0% (95% CI 3.8 to 10.3) in 1990-1991 to 3.1% (95% CI 1.7 to 4.6) in 1998-1999.

Among patients treated with BCS alone (no radiotherapy or tamoxifen), the five-year risk of local recurrence was 17.3% (95% CI 11.7 to 23.0) in 1990-1991 and 13.3% (95% CI 8.4 to 18.2) in 1998-1999; it was 8.5% (95% CI 1.4 to 15.7) and 6.1% (95% CI 3.2 to 8.9), respectively, for those treated with BCS plus radiotherapy. There were too few patients treated with tamoxifen in the early 1990s for reliable risk estimates across calendar years.

**Figure 3:**
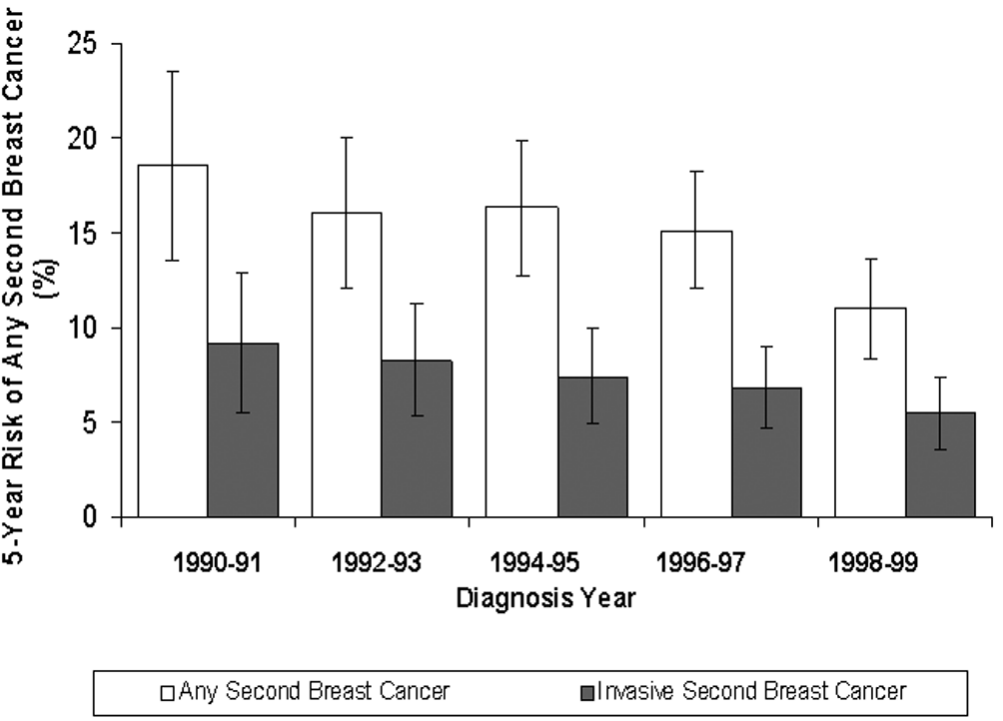
Five-year risk of any second breast cancer by diagnosis year. The white bars indicate the risk of any second breast cancer; the grey bars indicate the risk of an invasive second breast cancer. The length of the vertical line through the bar indicates the 95% confidence interval (CI).

The five-year risk of any second breast cancer event (ipsilateral, contralateral or regional/distant disease) decreased from 18.5% (95% CI 13.6 to 23.5) for patients diagnosed in 1990-1991 to 11.0% (95% CI 8.4 to 13.6) for patients diagnosed in 1998-1999 (Figure 3). The risk of any second invasive breast cancer decreased from 9.2% (95% CI 5.5 to 12.9) in 1990-1991 to 5.5% (95% CI 3.5 to 7.4) in 1998-1999.

Among patients treated with BCS alone (no radiotherapy or tamoxifen), the five-year risk of any second breast cancer was 20.8% (95% CI 14.7 to 26.9) in 1990-1991 and 15.2% (95% CI 10.0 to - 20.4) in 1998-1999; it was 15.4% (95% CI 6.1 to 24.7) and 11.2% (95% CI 7.4 to 15.0), respectively, for those treated with BCS plus radiotherapy.

The five-year risk of any contralateral disease slightly decreased from 3.8% (95% CI 1.4 to 6.2) for patients diagnosed in 1990-1991 to 3.2% (95% CI 1.7 to 4.7) for patients diagnosed in 1998-1999. Among patients treated with BCS alone (no radiotherapy or tamoxifen), the five-year risk of any contralateral disease was 2.9% (95% CI 0.4 to 5.4) in 1990-1991 and 1.9% (95% CI 0.04 to 3.8) in 1998-1999; it was 6.9% (95% CI 0.4 to 13.4) and 4.8% (95% CI 2.2 to 7.4), respectively, for those treated with BCS plus radiotherapy.

In Cox models accounting for time since diagnosis, the association between diagnosis year and risk of recurrence was modestly attenuated after adjustment for radiotherapy and tamoxifen (Table 2). Additional adjustment for age (using five-year age categories), race, family history of breast cancer, body mass index (BMI), method of detection of the initial DCIS, re-excision, or post-surgical mammogram in the three months after initial biopsy with DCIS did not materially change the relative risk estimates (not shown).

**Table 2:** Relative risks of local recurrence and of any second breast cancer at five years associated with diagnosis year

	Local recurrence		Any second breast cancer	
	**RR^a^**	**95% CI^b^**	**RR^a^**	**95% CI^b^**

**Univariate models**				

**Diagnosis year**				

1990-1991	1.0	(reference)	1.0	(reference)

1992-1993	0.7	0.5-1.0	0.8	0.6-1.1

1994-1995	0.8	0.6-1.2	0.9	0.6-1.2

1996-1997	0.7	0.5-1.0	0.7	0.5-0.9

1998-1999	0.4	0.3-0.6	0.4	0.3-0.6

	*P *for trend < 0.0001		*P *for trend < 0.0001	

**Adjuvant therapy**				

None (BCS^c ^only)	1.0	(reference)	1.0	(reference)

Radiotherapy	0.4	0.3-0.5	0.6	0.5-0.7

Tamoxifen	0.1	0.04-0.6	0.2	0.1-0.6

Radiotherapy+Tamoxifen	0.1	0.05-0.4	0.3	0.1-0.5

**Multivariable model (with treatment variables)**				

**Diagnosis year^d^**				

1990-1991	1.0	(reference)	1.0	(reference)

1992-1993	0.8	0.6-1.2	0.9	0.7-1.3

1994-1995	1.0	0.7-1.4	1.0	0.7-1.3

1996-1997	0.8	0.6-1.2	0.8	0.6-1.1

1998-1999	0.6	0.4-0.8	0.6	0.4-0.8

	*P *for trend = 0.008		*P *for trend = 0.0002	

**Adjuvant therapy**				

None (BCS^c ^only)	1.0	(reference)	1.0	(reference)

Radiotherapy	0.4	0.3-0.6	0.6	0.5-0.8

Tamoxifen	0.2	0.04-0.7	0.2	0.1-0.7

Radiotherapy+Tamoxifen	0.2	0.06-0.4	0.3	0.2-0.6

^a ^Relative Risk

^b ^Confidence Interval

^c ^Breast-conserving surgery

^d ^Model adjusted for adjuvant therapy (none, radiotherapy, tamoxifen)

### Trends in pathologic factors

Among the subgroup of DCIS patients included in the case-control study, there was very little change in tumor size across the diagnosis years 1990 to 2001. For example, the mean tumor size in calendar years 1990-1991, 1995-1996, and 2000-2001 was 11.5 mm (range 0.5 to 50 mm), 11.0 mm (range 1.0 to 37 mm), and 9.6 mm (range 0.5 to 34 mm) (*P *= 0.44), respectively. In contrast, the proportion of patients with high nuclear grade tumors decreased; in calendar years 1990-1991, 1995-1996, and 2000-2001, it was 46%, 28%, and 32%, respectively (*P *= 0.03). In addition, patients diagnosed in the later calendar years were less likely to have involved surgical margins. The proportion with involved surgical margins in calendar years 1990-1991, 1995-1996, and 2000-2001 were 15%, 10%, and 0%, respectively (*P *= 0.03). However, among those with clear surgical margins and with a known distance between the margin and the tumor, the proportion with a surgical margin of 10 mm or more did not appear to increase over time. For example, the proportion with a surgical margin of 10 mm or more in calendar years 1990-1991, 1995-1996, and 2000-2001 was 39%, 28%, and 30%, respectively (*P *= 0.37). The proportion of patients with comedo necrosis decreased only slightly; in calendar years 1990-1991, 1995-1996, and 2000-2001, it was 65%, 52%, and 55%, respectively (*P *= 0.14). The proportion of ER-negative tumors decreased somewhat; in calendar years 1990-1991, 1995-1996, and 2000-2001, it was 19%, 13%, and 11%, respectively (*P *= 0.44).

## Discussion

To our knowledge, this is the first study to provide information on changing risk of recurrence among DCIS patients treated with BCS in the community setting. Our findings suggest that DCIS patients diagnosed in the late 1990s had approximately half the risk of a recurrence at five years compared to patients diagnosed in the early 1990s. Furthermore, this was observed for recurrences that included both DCIS and invasive disease and when recurrences were restricted to invasive disease. The decline appeared to be only partially due to an increase in use of adjuvant radiotherapy and tamoxifen. Changes in pathologic practice during this period particularly with regard to margin evaluation also likely contributed to this decline.

Some limitations should be considered when interpreting our findings. While our study was drawn from the memberships of three large and diverse health plans, the results may not be generalizable to all geographic regions or community health care settings. However, the marked increases that we observed in adjuvant treatment with radiotherapy and tamoxifen over the study period are generally similar to those reported for DCIS patients in the overall U.S. population, based on the SEER Program Cancer Registries [2,8]. Conversely, treatment with tamoxifen in our health plans was somewhat lower than that reported for patients treated in National Comprehensive Cancer Network (NCCN) centers (for example, 34% in our health plans vs. 40 to 50% of NCCN patients in calendar years 2000-2001 [14]).

We did not have information on estrogen receptor (ER) status, a current indicator for treatment with tamoxifen [10], on the full patient cohort. We also did not have information on the full patient cohort on other pathologic features that may be associated with an increased risk of recurrence, such as large tumor size [20], high nuclear grade [21-24], involved or narrowly free surgical margins [21,25,26,31,32], and comedo necrosis [33]. However, pathology data from a central standardized review were available on a large subset of the cohort. While we were unable to adjust for pathology factors because cases and controls were matched on calendar year, our data indicated that there was little change in the distribution of tumor size over the calendar years of our study. It did appear that patients diagnosed and treated with BCS in the later calendar years were less likely to have high nuclear grade tumors and more likely to have clear surgical margins, although the proportion with wide margins did not seem to increase over time. They were also slightly less likely to have tumors with comedo necrosis or tumors that were ER-negative, although these changes were not statistically significant. While data are limited on potential changes over time in specific characteristics of DCIS diagnosed in the community setting, a similar slight decrease in the incidence of comedo DCIS has been observed in the national SEER data on DCIS [34]. The case-control design for our pathology data, with matching on diagnosis year, prevented us from directly examining the association between changes in histopathology and risk of recurrence over time. Nonetheless, our observation that several higher risk pathologic factors (such as high nuclear grade, comedo necrosis, involved margins) have decreased over time does suggest that changes in the proportion of patients with these factors may have at least in part, contributed to our observed decline in risk of recurrence among DCIS patients diagnosed and treated in the 1990s.

Published results are available from five prospective, randomized clinical trials of BCS treatment for DCIS [15,17-19,35,36]. In NSABP B-17, the five-year risk of ipsilateral breast cancer was 13.4% in women treated with lumpectomy plus radiotherapy, and 20.9% in women treated by lumpectomy alone [15]. Similar results were found in the EORTC 10853 and SweDCIS studies [16,17]. Women in the radiotherapy plus tamoxifen arm of the NSABP B-24 trial had fewer breast cancer events at five years than did those on radiotherapy plus placebo (8.2% vs. 13.4%, *P *= 0.0009) [15]. However, the UK/ANZ trial of BCS with or without radiotherapy and with or without tamoxifen found a reduction in ipsilateral DCIS, but not ipsilateral invasive disease, associated with tamoxifen therapy [19].

Our risk estimates for patients diagnosed in the early 1990s are fairly consistent with those reported in these clinical trials and from other population-based studies of DCIS [22,37], suggesting that, contrary to concerns [38], results from clinical trials of DCIS treatment appear to be generalizable to community practice. Our results are also consistent with observational studies that indicate that the use of these adjuvant therapies has increased substantially among DCIS patients treated with BCS [2,8,14]. As expected, we observed a substantial increase in use of adjuvant radiotherapy in 1992/1993, about the time when the first results of the NSABP B-17 trial were presented and published showing a 50% reduction in recurrence associated with use of radiotherapy among patients treated with lumpectomy [39]. In 1992 and 1993, we observed a slight increase in the use of tamoxifen, which was coincident with the middle of the accrual period for the NSABP B-24 trial of BCS and radiotherapy with or without adjuvant tamoxifen. The decrease in tamoxifen use in1994 may have been in response to reports that tamoxifen increases the risk of endometrial cancer [40]. We observed another increase in tamoxifen use in 1998 and 1999 when the first NSABP B-24 results showing a 50% reduction in recurrences were published [39]. Tamoxifen use for DCIS during the mid-to-late 1990s also may have been influenced by the Breast Cancer Prevention Trial (BCPT), which was designed to examine whether tamoxifen could prevent the development of breast cancer [41]. Women at high risk, but without a personal history of breast cancer (DCIS or invasive disease), were enrolled in the BCPT between 1992 and 1997.

While the age-adjusted incidence of a diagnosis of DCIS has increased during the 1990s [8], our results suggest that increasing use of adjuvant radiotherapy and tamoxifen during this same period contributed to a substantial decrease in the five-year risk of recurrence among DCIS patients treated with BCS in the community setting. Nonetheless, it should be noted that neither adjuvant radiotherapy nor radiotherapy plus tamoxifen have been shown to have a survival benefit and there is currently no consensus that all DCIS patients treated with BCS should receive adjuvant radiotherapy and tamoxifen [10,11]. Additional research is needed to confirm our findings, to examine whether the decline in risk of recurrence has continued for later diagnosis years, and to further examine the extent to which an increase in the proportion of patients with clear surgical margins or low or intermediate nuclear grade, or other factors, may also contribute to our observed decrease in risk of recurrence for DCIS patients treated with BCS.

## Conclusions

The marked increase in the 1990s in the use of radiotherapy and/or tamoxifen for DCIS patients treated with BCS suggests dissemination of findings from randomized trials into community practice. In our settings, treatment of DCIS in the late 1990s was associated with a five-year risk of recurrence of only 8% when BCS was used. If use of adjuvant radiotherapy and tamoxifen increased further after the late 1990s and early 2000s, as is likely given that adjuvant tamoxifen was only approved for DCIS patients in 2000, the five-year risk of recurrence may be even lower for DCIS patients diagnosed and treated more recently in the community setting. Further improvements in complete surgical excision and histologic assessment of DCIS lesions may also help to decrease recurrence rates.

## Abbreviations

BCPT: Breast Cancer Prevention Trial; BCS: breast-conserving therapy; BMI: body mass index; CRN: Cancer Research Network; DCIS: ductal carcinoma in situ; EORTC: European Organization for Research and Treatment of Cancer; ER: estrogen receptor; FDA: Federal Drug Administration; HMO: health maintenance organizations; HPHC: Harvard Pilgrim Health Care; KPNC: Kaiser Permanente of Northern California; KPSC: Kaiser Permanente of Southern California; NCCN: National Comprehensive Cancer Network; NSABP: National Surgical Adjuvant Breast and Bowel Project; SEER: Surveillance, Epidemiology, and End Results.

## Competing interests

The authors declare that they have no competing interests.

## Authors' contributions

LAH participated in the design of the study, acquisition of data, directed the data analysis, and drafted the manuscript. RH, LN, SWF, SJS, LCC, AMG and BP participated in the design of the study, acquisition of data, interpretation of results, and in writing the manuscript. NSA performed statistical analyses and participated in interpreting results. LA participated in the development of the study methods and coordinated data collection. CPQ participated in the study design, co-directed the data analysis, and participated in interpreting results. All authors read and approved the final manuscript.
